# Second-line agents in myositis: 1-year factorial trial of additional immunosuppression in patients who have partially responded to steroids

**DOI:** 10.1093/rheumatology/keu442

**Published:** 2014-11-27

**Authors:** Fowzia Ibrahim, Ernest Choy, Patrick Gordon, Caroline J. Doré, Alan Hakim, George Kitas, David Isenberg, Bridget Griffiths, Bryan Lecky, Kuntal Chakravarty, John Winer, Katalin Danko, Robert G. Cooper, Beverley White-Alao, David L. Scott

**Affiliations:** ^1^Department of Rheumatology, King’s College London, London, ^2^Cardiff Institute of Infection & Immunity, Cardiff University School of Medicine, Cardiff, ^3^MRC Clinical Trials Unit, ^4^Department of Rheumatology, Whipps Cross University Hospital, London, ^5^Rheumatology, Russells Hall Hospital, Dudley Group of Hospitals NHS Foundation Trust, Dudley, ^6^Department of Rheumatology, University College London Hospitals, London, ^7^Musculoskeletal Unit, Freeman Hospital, Newcastle, ^8^Department of Neurology, Walton Centre for Neurology and Neurosurgery, Liverpool, ^9^Department of Rheumatology, Queen’s Hospital, Romford, ^10^Department of Neurology, The Queen Elizabeth Hospital Neuroscience Centre, Birmingham, UK, ^11^Department of Internal Medicine, Medical University of Debrecen, Debrecen, Hungary and ^12^Rheumatic Diseases Centre, Salford Royal NHS Foundation Trust, Manchester, UK

**Keywords:** myositis and muscle disease, rheumatic diseases, DMARDs therapies, immunosuppressant therapies, clinical trials and methods, basic and clinical sciences, quality of life, psychology and social phenomena

## Abstract

**Objective.** Ciclosporin and MTX are used in idiopathic inflammatory myopathies (DM and PM) when patients incompletely respond to glucocorticoids. Their effectiveness is unproved in randomized controlled trials (RCTs). We evaluated their benefits in a placebo-controlled factorial RCT.

**Methods.** A 56-week multicentre factorial-design double-blind placebo-controlled RCT compared steroids alone, MTX (15–25 mg weekly) plus steroids, ciclosporin (1–5 mg/kg/day) plus steroids and all three treatments. It enrolled adults with myositis (by Bohan and Peter criteria) with active disease receiving corticosteroids.

**Results.** A total of 359 patients were screened and 58 randomized. Of the latter, 37 patients completed 12 months of treatment, 7 were lost to follow-up and 14 discontinued treatment. Patients completing 12 months of treatment showed significant improvement (*P* < 0.001 on paired *t*-tests) in manual muscle testing (14% change), walking time (22% change) and function (9% change). Intention to treat and completer analyses indicated that ciclosporin monotherapy, MTX monotherapy and ciclosporin/MTX combination therapy showed no significant treatment effects in comparison with placebo.

**Conclusion.** Neither MTX nor ciclosporin (by themselves or in combination) improved clinical features in myositis patients who had incompletely responded to glucocorticoids.

**Trial Registration**: International Standard Randomized Controlled Trial Number Register; http://www.controlled-trials.com/; ISRCTN40085050

## Introduction

Idiopathic inflammatory myositis (IIM) spans dermatomyositis and polymyositis. Initial treatment currently focuses on glucocorticoids, although there is little confirmatory randomized controlled trial (RCT) evidence [1]. Immune-modulating drugs are used when glucocorticoid-treated IIM patients have persisting disease or need steroid-sparing agents. RCT evidence supports giving these patients IVIG [2]. Open-label studies and case series suggest potential benefits from biologics (like rituximab) and conventional immunosuppressive agents [3]. Currently, RCT evidence for any of these treatments in adults is inconclusive or negative [4].

We addressed this uncertainty in a factorial RCT assessing two conventional immunosuppressive drugs. MTX and ciclosporin were used (either singly or in combination) in addition to glucocorticoids in IIM treatment. These therapies were selected because of positive observational evidence [3], differing modes of action, and positive findings in RA [5]. The trial studied glucocorticoid-treated patients with incomplete therapeutic responses and evidence of ongoing active disease.

## Methods

### Design

A 56-week double-blind 2 × 2 factorial RCT randomized patients to receive MTX (active or placebo) and ciclosporin (active or placebo).

### Patients

Male and female adults attending hospital outpatient clinics were enrolled.

*Inclusion criteria:* (i) definite IIM by Bohan and Peter criteria [6]; (ii) receiving glucocorticoids; (iii) active disease (muscle weakness 4/5 by manual muscle strength testing (MMT) in two or more muscle groups and functional deficit of one or more levels in one or more area of activities of daily living (using the functional rating scale); and (iv) willing and able to give informed consent.

*Exclusion criteria:* (i) under 18 years; (ii) inclusion body myositis and muscular dystrophies; (iii) unresponsive to 60 mg/day prednisolone for at least 4 weeks; (iv) family history of neuromuscular disease; (v) other serious disorders or contraindications (see supplementary Table S1, available at *Rheumatology* Online).

### Previous immunosuppressive treatments

Previous immunosuppressive treatments had been administered to 18 patients; of these, 8 had received MTX for a median of 2.1 years, and 2 had received ciclosporin for a median of 1.4 years. Patients had stopped these immunosuppressive treatments for a median of 0.3–3.6 years before entering the trial (see supplementary Table S2, available at *Rheumatology* Online).

The South-East Multicentre Research Ethics Committee approved Second Line Agents in Myositis (SELAM) (MREC Ref: 00/1/73). All enrolled patients gave written informed consent. The trial was registered with the UK Clinical Research Network and other relevant organizations (EudraCT number: 2004-001067-21; ISRCTN: 40085050; NIHR Porfolio ref: 2672).

### Treatments

MTX was given initially at 7.5 mg/week, increasing every 2 weeks by 2.5 mg to 15 mg/week. If there was persistent active disease, the dosage was increased by the supervising doctor to a maximum of 25 mg/week. Ciclosporin (microemulsion) was initiated at 1 mg/kg/day then increased where tolerated to a target of 5 mg/kg/day. If there was persistent active disease, the dosage was further increased to 10 mg/kg/day at the clinician’s discretion. Matched placebos were increased similarly. All patients remained on steroids, and the dosage of steroids was adjusted by the local researcher according to disease activity.

Patients continued analgesics (paracetamol or co-proxamol) or NSAIDs at standard dosages if needed. Other treatments (e.g. antihypertensives) were continued as needed. All patients received folic acid (5 mg/week). Patients on high-dose CS received appropriate bone protection.

### Outcomes

Patients were assessed at baseline and 12, 28, 40 and 56 weeks. The primary outcome measure was MMT at 56 weeks [7]. Secondary outcome assessments included the functional rating scale (FRS) [8], 30-m walking time (WT), creatine kinase (normal laboratory range up to 150 IU/ml), ESR (normal laboratory range up to 20 mm/h), treatment withdrawals, and adverse reactions.

### Sample size

Sample size was based on previous studies suggesting that MTX and ciclosporin improve MMT scores by 10% [4]. As RA trials show that ciclosporin and MTX have additive effects, we used a factorial 2 × 2 design [5]. Assuming MTX and ciclosporin groups had effect sizes of 1 (10% improvement with 10% s.d. %), an adjusted effect size based on intermediate dispersion for the four treatment groups (f) was 0.4. Detecting differences at the 5% level with 80% power required 18 per group (72 patients in total). Recruitment was slower than anticipated, and when 58 patients had been recruited, the Data Monitoring and Ethics Committee recommended that no further patients were enrolled as a positive outcome appeared increasingly unlikely.

### Randomization and allocation concealment

Patients were randomly allocated to receive steroids alone, steroids plus MTX, steroids plus ciclosporin, or steroids plus MTX plus ciclosporin. Randomization was stratified by centre, diagnosis (PM or DM) and by previous treatment (ciclosporin or MTX). Randomization numbers were assigned chronologically by centre after successful screening. Metrologists and investigators were unaware of the allocation sequence. Treatment assignments were in a locked cabinet in the coordinating centre pharmacy. Trial medication (MTX and ciclosporin) and identical placebos were pre-packed in identical containers. They were consecutively numbered for patients by centre according to the randomization schedule. Each patient received treatments in pre-packed containers.

### Statistical methods

Data management and analyses used Stata (version 12.0, StataCorp, College Station, TX, USA). Baseline characteristics were summarized by randomized group. Descriptive summary statistics were presented as mean (s.d.) for continuous normally distributed variables, as median and interquartile range for other continuous variables, and as frequency and percentage for categorical variables.

All participants had observations at baseline. Missing follow-up data were imputed by multiple imputations using multivariate normal regression, using an iterative Markov chain Monte Carlo method to impute missing values with 20 cycles. The 20 datasets were combined using Rubin’s rules [9–11]. Estimates and standard errors are presented as combined ones. A linear mixed model was used to analyse the primary and secondary outcomes. Random intercepts and slopes were fitted for each patient, along with a random effect of centre.

Intention-to-treat analyses were performed on the imputed data from all randomized patients. Completer analyses were performed on patients who completed 12 months. An interaction between treatment effects and time was included. The estimates are presented as coefficients with 95% CI; robust standard errors were used to take account of the clustering effect of different geographical regions in the estimation of standard errors and *P*-values. The estimates were adjusted for age, gender, ethnicity, diagnosis (DM/ PM), and previous treatment with MTX or ciclosporin. Statistical significance was determined at the 5% level using a two-sided *P*-value.

## Results

**Fig. 1:**
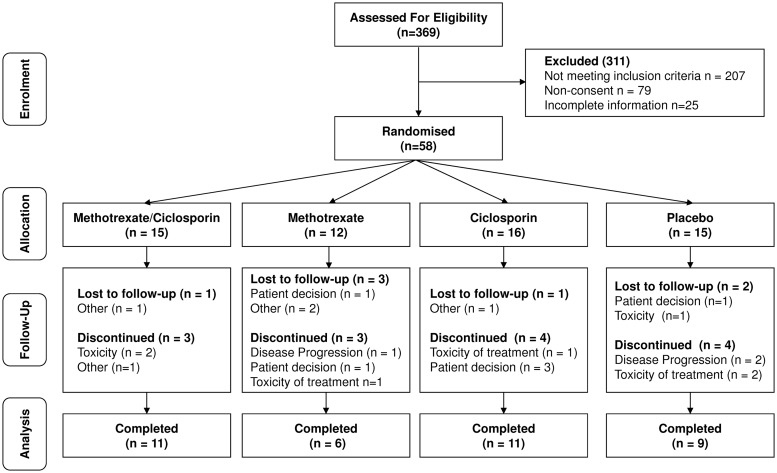
CONSORT flowchart for the SELAM trial

A total of 369 patients were screened and 58 patients were randomized. Screened patients who were not randomized comprised 207 patients who did not meet the inclusion criteria, 79 patients who did not consent and 25 with only incomplete information about their disease. Twelve to sixteen patients were randomized to each group (Fig. 1). Demography and baseline disease activity assessments were similar for each group, as was previous immunosuppressive treatment (supplementary Tables S2 and S3, available at *Rheumatology* Online).

Of the 58 randomized patients, 37 completed 12 months of treatment, 7 were lost to follow-up and 14 discontinued treatment. Of the latter, three patients withdrew or stopped treatment due to disease progression, six due to toxicity, four due to patient decision and one due to other reasons. Mean (s.d.) doses of glucocorticoid administered during the trial was 26.77 (s.d. 23.38) mg.

In the patients who completed 12 months of treatment, mean MMT increased from 64 to 73 (14% improvement), WT decreased from 36 to 28 (22% improvement) and FRS increased from 33 to 36 (9% improvement). These changes were all significant on paired *t*-tests (*P* = 0.0001, 0.0064, 0.0009, respectively). Improvements over 12 months were correlated; for example, improvements in FRS were related to improvements in MMT (Spearman’s correlation 0.59) and WT (Spearman’s correlation − 0.29). Initial and final change scores for each group are shown in supplementary Table S4, available at *Rheumatology* Online.

There was no evidence of significant treatment effects in either the intention-to-treat analysis (Table 1) or the completer analysis (supplementary Table S5, available at *Rheumatology* Online). In comparison with placebo therapy, ciclosporin monotherapy, MTX monotherapy and ciclosporin–MTX combination therapy all showed no evidence of significant benefits in unadjusted or adjusted analyses. Furthermore, no significant main effects of treatment were found when comparing MTX with MTX-placebo or ciclosporin with ciclosporin-placebo (supplementary Fig. S1, available at *Rheumatology* Online). There was no evidence that immunosuppressive treatment reduced glucocorticoid use: the mean daily prednisolone dose at the end of the trial comprised 18 mg in the placebo group compared with 22–26 mg in the various treatment groups.

**Table 1:** Comparison of outcomes at 12 months between treatment groups in an intention-to-treat analysis

		Unadjusted		Adjusted^a^	
Outcome	Group	Coefficients (95% CI)	*P*-value	Coefficients (95% CI)	*P*-value
Manual muscle testing	MTX/Ciclo	−2.72 (−8.60, 3.17)	0.365	−1.02 (−5.96, 3.92)	0.686
	MTX	1.03 (−4.88, 6.94)	0.732	0.17 (−4.35, 4.69)	0.942
	Ciclo	−1.13 (−7.50, 5.25)	0.729	0.42 (−3.82, 4.66)	0.845
30-m walk	MTX/Ciclo	5.03 (−6.23, 16.29)	0.381	3.31 (−6.79, 13.42)	0.520
	MTX	6.95 (−8.22, 22.12)	0.369	8.34 (−3.35, 20.03)	0.162
	Ciclo	8.21 (−3.21, 19.63)	0.159	8.77 (−1.22, 18.76)	0.085
Function, FRS	MTX/Ciclo	−1.91 (−4.55, 0.74)	0.158	−1.58 (−4.06, 0.91)	0.214
	MTX	−2.04 (−4.60, 0.52)	0.118	−2.43 (−4.92, 0.05)	0.055
	Ciclo	−1.43 (−4.02, 1.16)	0.278	−1.00 (−3.39, 1.39)	0.412
Creatine phosphokinase	MTX/Ciclo	−426 (−963, 111)	0.120	−365 (−793, 62)	0.094
	MTX	−455 (−973, 62)	0.085	−371 (−756, 15)	0.060
	Ciclo	−360 (−889, 170)	0.184	−378 (−859, 104)	0.124
ESR	MTX/Ciclo	−1.99 (−11.77, 7.79)	0.69	−3.04 (−11.56, 5.49)	0.484
	MTX	0.99 (−12.03, 14.00)	0.882	0.78 (−11.88, 13.44)	0.904
	Ciclo	5.60 (−4.37, 15.57)	0.271	5.09 (−4.42, 14.60)	0.294

^a^Adjusted for age, gender, ethnicity and diagnosis (DM/PM); placebo is the reference group; Ciclo: ciclosporin; FRS: functional rating scale.

Adverse events were reported by 50 patients (MTX–ciclosporin 12, MTX 10, ciclosporin 15, placebo 13), although only 14 withdrew because of this (Fig. 1). The most commonly seen adverse events (>5% of patients) comprised: musculoskeletal (MTX–ciclosporin 1, MTX 1, ciclosporin 1, placebo 2); gastrointestinal (MTX–ciclosporin 4, MTX 4, placebo 5); and respiratory (MTX–ciclosporin 3, MTX 2, ciclosporin 3, placebo 1).

## Discussion

SELAM showed that MTX monotherapy, ciclosporin monotherapy or ciclosporin–MTX combination therapy) improved disease activity in adult IIM patients with incomplete response to glucocorticoids. Continuing to use these drugs in such patients is questionable. They have significant toxicities in many rheumatic diseases without evidence of efficacy in this clinical setting. Although IIM patients enrolled in SELAM showed significant improvements (9–22%) in key 12-month clinical outcomes, there was no indication that immunosuppressive therapy influenced these changes.

A recent study assessed prednisolone alone or combined with MTX and ciclosporin in JDM. The times to inactive disease and major therapeutic changes were less in the combination groups compared with steroid monotherapy [12]. However, both outcomes incorporated the physician’s opinion of disease activity and could be influenced by the study’s open-label design; consequently, the findings must be viewed with caution. The JDM study initiated immunosuppressive therapy with steroids, whereas our study assessed its efficacy in active disease despite steroid therapy. It is possible that initial cytotoxic use may be more effective than delayed treatment.

SELAM has several potential limitations. First, as it was placebo-controlled, clinicians may have been unwilling to enrol patients with severe IIM. Only a minority of screened patients were enrolled in SELAM. Although detailed information on screen failures could not be collected for ethical reasons, our clinical impression was that many were taking additional MTX, ciclosporin or AZA that could not be temporarily stopped. Secondly, the immunosuppressive treatment may have been too conservative; more intensive treatment might be effective. In addition, a few patients had received previous immunosuppressive therapy, although as outlined in supplementary Table S2, available at *Rheumatology* Online, this is unlikely to have had a significant impact. Thirdly, the outcome measures might have been too insensitive, particularly as SELAM predated the standardization of IIM outcomes [13]. Fourthly, some muscle weakness may have been irreversible due to muscle damage or steroid myopathy. Fifthly, treating all IIM patients similarly may be inappropriate, and there may be different responses depending on patients’ autoantibody profiles [14]. Sixthly, Bohan and Peter’s criteria have been used for over 35 years and alternative criteria exist; using these might have identified patients more likely to respond to immunosuppressive therapy. Seventhly, only 37 of 58 patients (64%) completed 12 months of treatment; although withdrawals were not especially high for a 12-month placebo-controlled trial, they might dilute the positive impacts of treatment. Finally, the trial was relatively small and enrolment was stopped by the Data Monitoring Committee (DMC) before the planned sample size was reached. Small trials can miss treatment effects in specific patient subgroups. Although the absence of evidence for an overall treatment effect makes it unlikely that studying more patients would have changed the findings, alternative approaches to patient selection might have produced positive findings.

There is strong evidence that some additional treatments benefit IIM patients who are incomplete responders to glucocorticoids. Examples include IVIG [2] and creatine supplements combined with intensive exercise [15]. Biologics like rituximab, despite initially promising results [16–18], have yet to deliver clear therapeutic benefits when studied in RCTs [19]. The failure of conventional immunosuppressives to benefit IIM patients who have incompletely responded to glucocorticoids does not mean that these agents are ineffective, only that the particular treatment paradigm studied in SELAM appears inappropriate. Immunosuppressives could be effective as initial combinations, although as some patients respond to steroids alone the benefit of such an approach is uncertain.

Future research needs to identify effective treatments in IIM patients who fail to respond to glucocorticoids and to define optimal initial treatments. Potential novel treatments include eculizumab [20] and abatacept (being evaluated in the ARTEMIS trial—http://clinicaltrials.gov/ct2/show/NCT01315938). The negative results in SELAM highlight the need for further RCTs in IIM.

**Rheumatology key messages**

- Patients with active inflammatory myositis taking oral steroids improve over time.
- There is no evidence that adding MTX or ciclosporin benefits patients with active inflammatory myositis taking oral steroids.
- In patients with active inflammatory myositis taking oral steroids, there is no evidence that combining MTX and ciclosporin is beneficial.

## Supplementary Material

Supplementary Data
