# Supplementary material for: Second-line agents in myositis: 1-year factorial trial of additional immunosuppression in patients who have partially responded to steroids
Source: Rheumatology (Oxford). 2014 Nov 27;54(6):1050–5. doi: 10.1093/rheumatology/keu442 (PMC4476843; doi:10.1093/rheumatology/keu442)
Supplement: Supplementary Data [file supp_54_6_1050__index.html]

Second-line agents in myositis: 1-year factorial trial of additional immunosuppression in patients who have partially responded to steroids — Second-line agents in myositis: 1-year factorial trial of additional immunosuppression in patients who have partially responded to steroids — Supplementary Data 

# Second-line agents in myositis: 1-year factorial trial of additional immunosuppression in patients who have partially responded to steroids

## Supplementary Data

files

**Files in this Data Supplement:**

- Supplementary Data - docx file
