# Supplementary material for: Second-line agents in myositis: 1-year factorial trial of additional immunosuppression in patients who have partially responded to steroids
Source: Rheumatology (Oxford). 2014 Nov 27;54(6):1050–5. doi: 10.1093/rheumatology/keu442 (PMC4476843; doi:10.1093/rheumatology/keu442)
Supplement: Supplementary Data [file supp_keu442_rhe-13-1329-File003.docx]

**Supplementary Data**

**Supplementary Table S1. Exclusion Criteria**

| 1. Age <18 years 2. Inclusion body myositis and muscular dystrophies. If the existing muscle biopsy is not definitely diagnostic of myositis, or if the muscle biopsy is unclear or suffering from a sampling error, the results will be reviewed by an independent pathologist, or the muscle biopsy will be repeated 3. Unresponsive to high dosage corticosteroids, equivalent to 60mg per day of prednisolone taken for a period of at least 4 weeks 4. Family history of any neuromuscular disease 5. Uncorrected disorders of thyroid function 6. Uncorrected disorders of calcium metabolism 7. Any significant degree of hepatic dysfunction 8. Treatment with methotrexate, azathioprine or ciclosporin within the last 4 weeks, or treatment with leflunomide within the last 8 weeks 9. Other serious uncontrolled medical disorders (e.g. Hepatic failure and cardiac failure) 10. Concurrent malignant disease 11. Individuals of child bearing/fathering potential who are not taking adequate contraceptive protection and continue with this for at least 6 months after stopping methotrexate therapy 12. Contra-indications for ciclosporin therapy: significant renal impairment (persistently elevated serum creatinine); concomitant treatment with drugs of known nephritic potential excluding NSAIDs; uncontrolled hypertension with diastolic blood pressures consistently >95mmhg or systolic blood pressure consistently >160mmhg despite anti-hypertensive medication 13. Neutrophil count <1.0 x 1012/dl or platelet count <100 x 1012/dl 14. Patients with systemic lupus erythematosus, scleroderma, Sjogren's syndrome or rheumatoid arthritis who currently require treatment for these conditions. Patients who have been previously diagnosed with these conditions but in whom the condition is no longer active will be eligible for the SELAM trial 15. Chronic alcoholism 16. Drug-induced myositis 17. Patients with life-threatening complications of inflammatory myositis including ventilator failure and severe bulbar involvement requiring urgent aggressive immunosuppression. |
| --- |

**Supplementary Table S2: Previous Immunosuppressive Treatments**

| **Prior Treatment** | **Methotrexate-Ciclosporin** | **Methotrexate** | **Ciclosporin** | **Placebo** | **Total** | **Median Duration Treatment (Years)** | **Median Treatment Discontinuation (Years)** |
| --- | --- | --- | --- | --- | --- | --- | --- |
|  | *n=15* | *n=12* | *n=16* | *n=15* | *n=58* |  |  |
| Methotrexate (%) | 2 | 1 | 2 | 3 | 8 (14%) | 2.1 | 0.3 |
| Ciclosporin (%) | 0 | 1 | 1 | 0 | 2 (3%) | 1.4 | 3.6 |
| Cyclophosphamide (%) | 0 | 1 | 2 | 2 | 5 (9%) | 0.4 | 3.0 |
| Intravenous-gammaglobulin (%) | 1 | 1 | 1 | 1 | 4 (7%) | 0.5 | 2.1 |
| Chlorambucil (%) | 0 | 1 | 0 | 0 | 1 (2%) | 1.5 | 1.6 |

18 patients had received previous immunosuppressive treatments. 8 had received methotrexate for a median of 2.1 years. 2 had received ciclosporin for a median of 1.4 years. Patients had stopped these immunosuppressive treatments for a median of 0.3 to 3.6 years before entering the trial. The patients had been treated in routine practice settings without standardised information on overall dosages, adherence to therapy or response to treatment being regularly recorded. However, the relatively prolonged treatment periods in some patients indicate the supervising clinicians considered they were beneficial. One patient had received plasmaphersis as a non-drug immunsuppressive. All patients stopped treatment for at least one month before trial entry, reflecting the approach taken in published trials of disease modifying drugs^1^. Only 4 patients had such a short interval and the median period of stopping treatment was 1.6 years. Including patients previously treated with methotrexate has been used in trials of combination treatment in rheumatoid arthritis by O’Dell et al^2,3^. Restarting methotrexate is effective in rheumatoid arthritis, as shown in follow-up of the classic initial cross-over trial^4^. There is no comparable data on methotrexate use in trials in inflammatory myositis.

1. Emery P, Breedveld FC, Lemmel EM, Kaltwasser JP, Dawes PT, Gömör B et al. A comparison of the efficacy and safety of leflunomide and methotrexate for the treatment of rheumatoid arthritis. Rheumatology 2000; 39: 655-65.
2. O'Dell JR, Leff R, Paulsen G, Haire C, Mallek J, Eckhoff PJ et al. Treatment of rheumatoid arthritis with methotrexate and hydroxychloroquine, methotrexate and sulfasalazine, or a combination of the three medications: results of a two-year, randomized, double-blind, placebo-controlled trial. Arthritis Rheum 2002; 46: 1164-70.
3. O'Dell JR, Haire CE, Erikson N, Drymalski W, Palmer W, Eckhoff PJ, et al. Treatment of rheumatoid arthritis with methotrexate alone, sulfasalazine and hydroxychloroquine, or a combination of all three medications. N Engl J Med 1996; 334: 1287-91.
4. Weinblatt ME, Trentham DE, Fraser PA, Holdsworth DE, Falchuk KR, Weissman BN, et al. Long-term prospective trial of low-dose methotrexate in rheumatoid arthritis. Arthritis Rheum 1988; 31: 167-75.

**Supplementary Table S3: Baseline Characteristics of Randomised Patients**

|  | |  | **Methotrexate/Ciclosporin** | **Methotrexate** | **Ciclosporin** | **Placebo** |
| --- | --- | --- | --- | --- | --- | --- |
|  | |  | *n=15* | *n=12* | *n=16* | *n=15* |
| Females/Males (number, %) | | | 11 (73%)/4 (27%) | 10 (83%)/2 (17%) | 10 (63%)/6 (38%) | 9 (60%)/6 (40%) |
| White European/Other (number, %) | | | 13 (87%)/2 (13%) | 11 (92%)/1 (8%) | 10 (63%)/6 (38%) | 12 (80%)/3 (20%) |
| Age in years (mean, SD) | | | 55 (13) | 50 (17) | 48 (15) | 49 (11) |
| Disease duration in years (mean, SD) | | | 2.03 (2.78) | 1.83 (3.12) | 2.02 (4.82) | 2.57 (4.33) |
| Dermatomyositis/Polymyositis (number, %) | | | 5 (36%)/9 (64%) | 7 (58%)/5 (42%) | 6 (38%)/ 10 (63%) | 8 (53%)/7 (47%) |
| Height in cm (mean, SD) | | | 163 (12.1) | 167 (5.3) | 168(5.1) | 167 (8.4) |
| Weight in kg (mean, SD) | | | 72 (13.6) | 74 (14.5) | 77 (25.2) | 76 (14.3) |
| Glucocorticoid dose in mg/day (mean, range) | | | 23 (12-40) | 24 (6-58) | 30 (13-55) | 22 (4-75) |
| Baseline Assessments | *ESR mm/hr median (IQR)* | | 9 (6-22) | 8 (5-44) | 19 (9-26) | 11 (6-29) |
|  | *Global assessment* | | 58 (42 80) | 57 (41-68) | 55 (44-74) | 48 (25-67) |
|  | *Manual muscle test (mean, SD)* | | 63 (7) | 68 (9) | 66 (13) | 65 (10) |
|  | *30 meter walk (median, IQR)* | | 25 (22-40) | 29 (23-36) | 30 (20–29) | 21 (20-29) |
|  | *FRS (mean, SD)* | | 32 (6) | 32 (4) | 32 (5) | 35 (4) |
|  | *Creatine Phosphokinase (median, IQR)* | | 310 (96-696) | 104 (58-157) | 326 (88-1083) | 309 (45-1863) |

SD (standard deviation); IQR (interquartile range); FRS (Functional Rating Scale)

**Supplementary Table S4: Initial, Final And Change Scores In Main Outcome Assessments By Trial Group**

| **Outcome** |  | **Methotrexate/Ciclosporin** | **Methotrexate** | **Ciclosporin** | **Placebo** |
| --- | --- | --- | --- | --- | --- |
|  |  | *n=15* | *n=12* | *n=16* | *n=15* |
|  |  | *Mean (95%CI)* | *Mean (95%CI)* | *Mean (95%CI)* | *Mean (95%CI)* |
| **Manual Muscle Testing** | *Initial* | 62.85 (58.96 , 66.74) | 67.57 (62.40 , 72.75) | 66.24 (59.97 , 72.51) | 65.05 (59.70 , 70.40) |
|  | *One year* | 71.41 (66.18 , 76.63) | 75.27 (69.98 , 80.55) | 72.13 (65.40 , 78.87) | 78.43 (73.69, 83.16) |
|  | *Change*  *(One year -initial)* | 8.56 (2.94, 14.17) | 7.70 (1.36 , 14.04) | 5.90 (-0.60 , 12.40) | 13.38 (8.39 , 18.37) |
| **30 Metre Walk** | *Initial* | 34.65 (23.67, 45.64) | 35.27 (22.06 , 48.49) | 37.08 (24.95 , 49.20) | 30.58 (19.64 , 41.51) |
|  | One year | 28.45 (19.46 , 37.44) | 30.77 (17.15 , 44.39) | 31.68 (22.82 , 40.53) | 24.94 (16.55 , 33.34) |
|  | *Change*  *(One year -initial)* | -6.20 (-15.29 , 2.89) | -4.50 (-13.96 , 4.95) | -5.40 (-12.67 , 1.87) | -5.63 (-13.35 , 2.08) |
| **Function (FRS)** | *Initial* | 31.70 (28.19 , 33.95) | 31.83 (29.79 , 33.88) | 32.19 (29.65 , 34.72) | 34.60 (32.68 , 36.52) |
|  | *One year* | 35.09 (32.83 , 37.35) | 34.73 (32.21 , 37.26) | 34.97 (32.46 , 37.48) | 36.26 (34.17 , 38.34) |
|  | *Change*  *(One year -initial)* | 4.02 (0.91 , 7.12) | 2.90 (0.70 , 5.11) | 2.78 (-0.02 , 5.58) | 1.66 (-0.58 , 3.90) |
| **Creatine Phosphokinase** | *Initial* | 436.57 (-161.81 , 1,034.96) | 100.33 (32.96 , 167.71) | 303.75 (105.95 , 501.55) | 667.87 (43.21 , 1,292.53) |
|  | *One year* | 203.74 (-23.79 , 431.27) | 148.98 (18.71 , 279.26) | 258.19 (75.61 , 440.77) | 684.30 (78.37 , 1,290.24) |
|  | *Change*  *(One year -initial)* | -232.83 (-757.32 , 291.66) | 48.65 (-63.41 , 160.71) | -45.56 (-192.06 , 100.94) | 16.44 (-92.90 , 125.77) |
| **ESR** | *Initial* | 13.63 (5.95 , 21.31) | 15.85 (3.00 , 28.70) | 19.31 (11.88 , 26.74) | 13.93 (5.87 , 21.99) |
|  | *One year* | 14.05 (5.60 , 22.50) | 18.24 (7.25 , 29.22) | 22.41 (15.81 , 29.01) | 17.46 (7.67 , 27.25) |
|  | *Change*  *(One year -initial)* | 0.41 (-9.51 , 10.34) | 2.39 (-4.81 , 9.59) | 3.10 (-2.10 , 8.29) | 3.53 (-2.71 , 9.76) |

**Supplementary Table S5: Comparison of Outcomes at 12 Months between Treatment Groups in a Completer Analysis**

| **Outcome** | **Group** | **Unadjusted** | | **Adjusted^*^** | |
| --- | --- | --- | --- | --- | --- |
|  |  | *Coefficients (95% CI)* | *p-value* | *Coefficients (95% CI)* | *p-value* |
| **Manual Muscle Testing** | *Mtx/Ciclo* | -1.69 (-9.27, 5.89) | 0.662 | -3.69 (-10.06, 2.67) | 0.255 |
|  | *Mtx* | 3.36 (-3.61, 10.33) | 0.344 | 5.71 (-1.43, 12.85) | 0.117 |
|  | *Ciclo* | 1.53 (-6.85, 9.90) | 0.721 | 1.81 (-4.20, 7.82) | 0.555 |
|  |  |  |  |  |  |
| **30 Metre Walk** | *Mtx/Ciclo* | 0.25 (-14.17, 14.66) | 0.973 | 5.79 (-5.34, 16.93) | 0.308 |
|  | *Mtx* | 3.83 (-22.11, 29.77) | 0.772 | 9.41 (-8.32, 27.14) | 0.298 |
|  | *Ciclo* | 4.82 (-11.37, 21.01) | 0.560 | 12.21 (-2.50, 26.92) | 0.104 |
|  |  |  |  |  |  |
| **Function (FRS)** | *Mtx/Ciclo* | -2.87 (-5.70, -0.04) | 0.047 | -3.27 (-5.32, -1.22) | 0.002 |
|  | *Mtx* | -2.08 (-4.99, 0.83) | 0.162 | -1.96 (-4.24, 0.31) | 0.090 |
|  | *Ciclo* | -2.08 (-4.84, 0.67) | 0.139 | -2.24 (-4.84, 0.37) | 0.092 |
|  |  |  |  |  |  |
| **Creatine Phosphokinase** | *Mtx/Ciclo* | -284 (-1,017, 448) | 0.447 | -423 (-1,220, 375) | 0.299 |
|  | *Mtx* | -406 (-1,106, 294) | 0.256 | -232 (-721, 256) | 0.351 |
|  | *Ciclo* | -288 (-1,017, 441) | 0.438 | -422 (-1,247, 402) | 0.315 |
|  |  |  |  |  |  |
| **ESR** | *Mtx/Ciclo* | -5.22 (-13.49, 3.06) | 0.216 | -6.35 (-12.86, 0.16) | 0.056 |
|  | *Mtx* | -4.51 (-16.62, 7.60) | 0.465 | -8.90 (-22.08, 4.29) | 0.186 |
|  | *Ciclo* | 5.65 (-4.94, 16.24) | 0.296 | 2.66 (-7.04, 12.35) | 0.591 |

^*^Adjusted for age, gender, ethnicity, diagnosis (Dermatomyositis/ Polymyositis) and previous treatment with Methotrexate or Ciclosporin; Placebo is the reference group; CI confidence intervals

**Supplementary Figure S1. Mean Initial and 12 Month Manual Muscle Testing Scores With Methotrexate Or Ciclosporin**


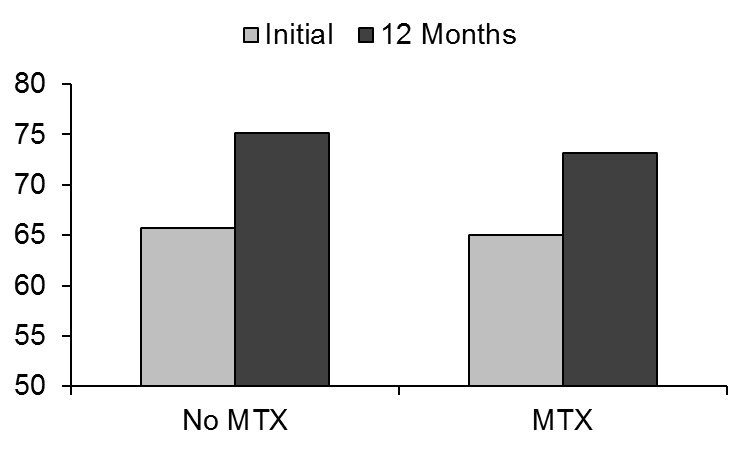

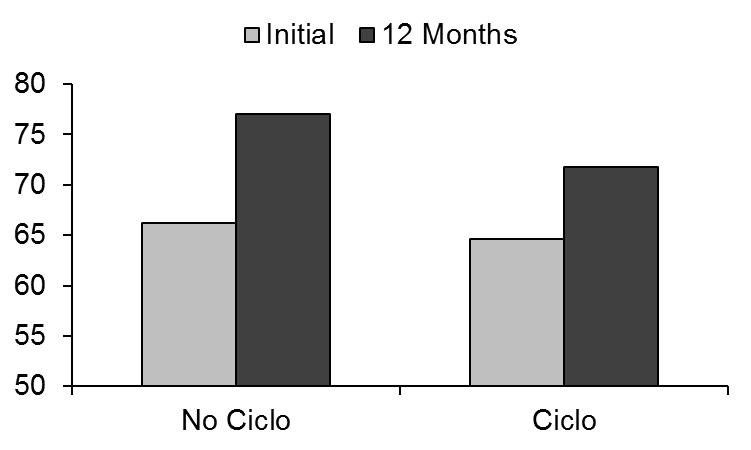


**Completers**


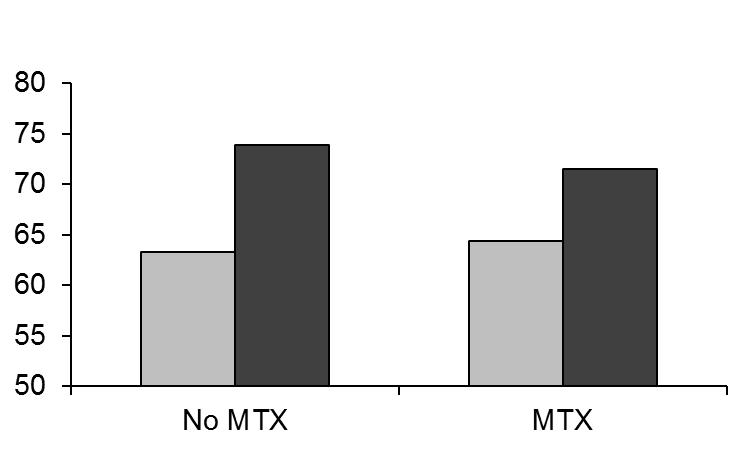

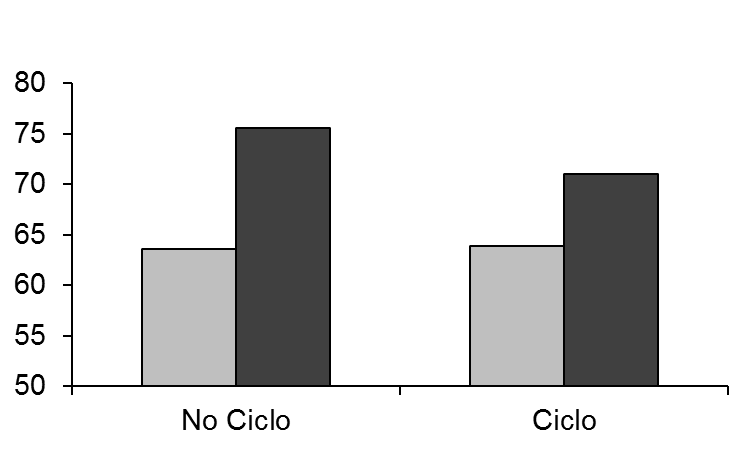


**Intention To Treat**
